# Virtual screening, identification and *in vitro* validation of small molecule GDP-mannose dehydrogenase inhibitors†

**DOI:** 10.1039/d3cb00126a

**Published:** 2023-08-29

**Authors:** Jonathan P. Dolan, Sanaz Ahmadipour, Alice J. C. Wahart, Aisling Ní Cheallaigh, Suat Sari, Chatchakorn Eurtivong, Marcelo A. Lima, Mark A. Skidmore, Konstantin P. Volcho, Jóhannes Reynisson, Robert A. Field, Gavin J. Miller

**Affiliations:** a Lennard-Jones Laboratory, School of Chemical & Physical Sciences, Keele University Keele Staffordshire ST5 5BG UK g.j.miller@keele.ac.uk; b Centre for Glycoscience, Keele University Keele Staffordshire ST5 5BG UK; c Hornbeam Building, School of Pharmacy & Bioengineering, Keele University Keele Staffordshire ST5 5BG UK; d School of Life Sciences, Keele University Keele Staffordshire ST5 5BG UK; e Department of Chemistry & Manchester Institute of Biotechnology, The University of Manchester 131 Princess Street Manchester M1 7DN UK; f Hacettepe University, Faculty of Pharmacy, Department of Pharmaceutical Chemistry 06100 Ankara Turkey; g Department of Pharmaceutical Chemistry, Faculty of Pharmacy, Mahidol University 447 Si Ayutthaya Road Ratchathewi Bangkok 10400 Thailand; h N. Vorozhtsov Novosibirsk Institute of Organic Chemistry, Siberian Branch of the Russian Academy of Sciences 630090 Novosibirsk Russia

## Abstract

Upon undergoing mucoid conversion within the lungs of cystic fibrosis patients, the pathogenic bacterium *Pseudomonas aeruginosa* synthesises copious quantities of the virulence factor and exopolysaccharide alginate. The enzyme guanosine diphosphate mannose dehydrogenase (GMD) catalyses the rate-limiting step and irreversible formation of the alginate sugar nucleotide building block, guanosine diphosphate mannuronic acid. Since there is no corresponding enzyme in humans, strategies that could prevent its mechanism of action could open a pathway for new and selective inhibitors to disrupt bacterial alginate production. Using virtual screening, a library of 1447 compounds within the Known Drug Space parameters were evaluated against the GMD active site using the Glide, FRED and GOLD algorithms. Compound hit evaluation with recombinant GMD refined the panel of 40 potential hits to 6 compounds which reduced NADH production in a time-dependent manner; of which, an usnic acid derivative demonstrated inhibition six-fold stronger than a previously established sugar nucleotide inhibitor, with an IC_50_ value of 17 μM. Further analysis by covalent docking and mass spectrometry confirm a single site of GMD alkylation.

## Abstract 2

Identification of the first small molecule inhibitor for the guanosine diphosphate mannose dehydrogenase from *Pseudomonas aeruginosa*.

## Graphical abstract

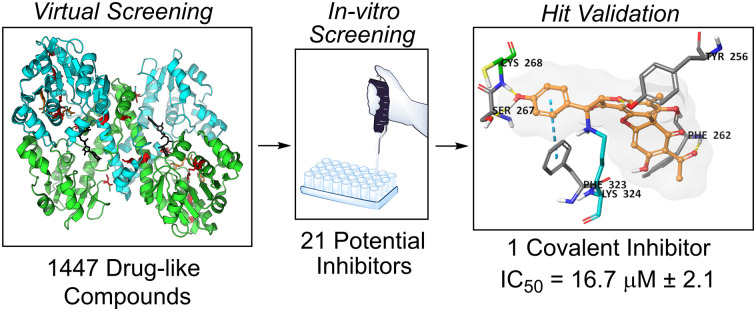

## Introduction

**Fig. 1:**
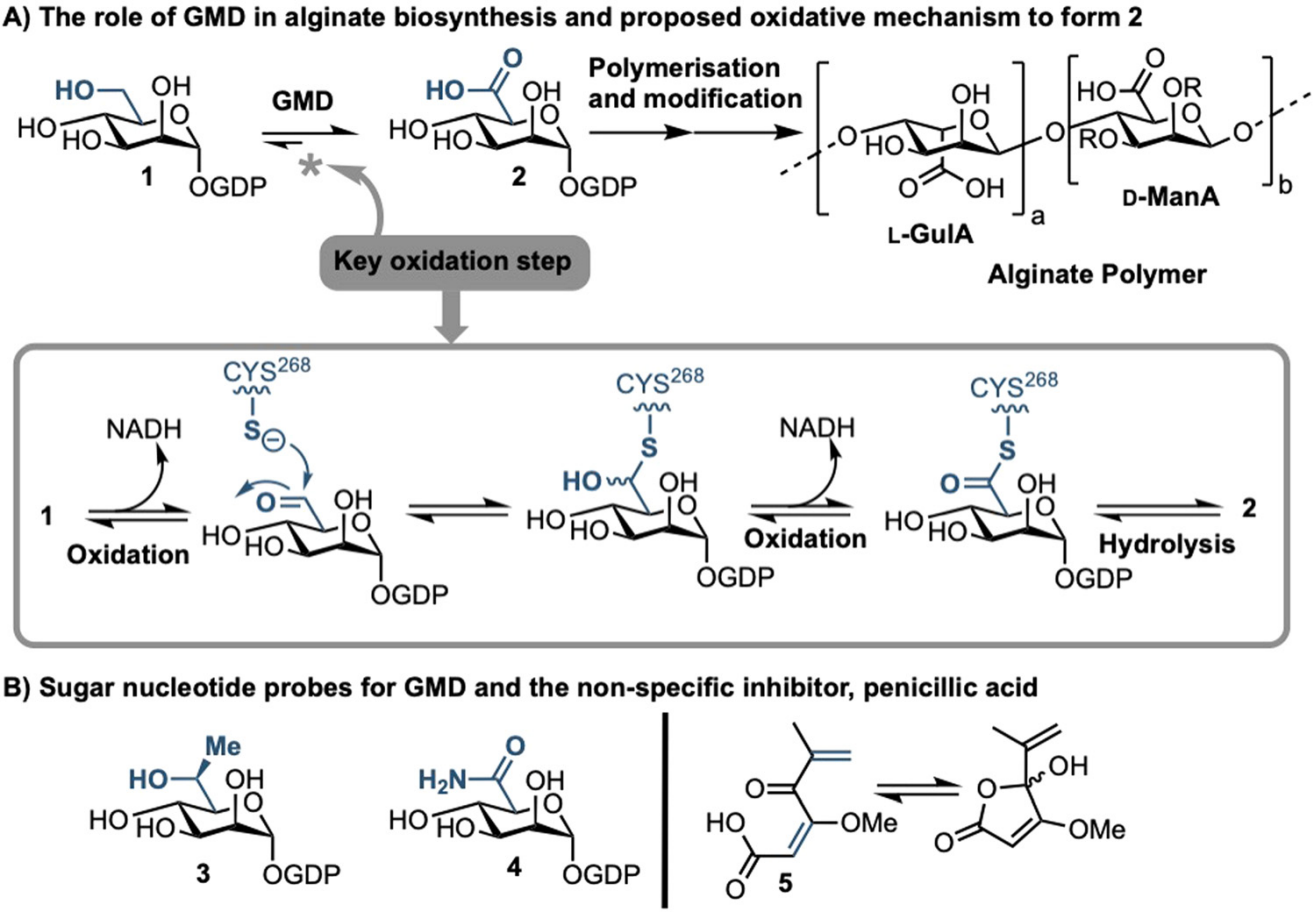
(A) Role of GMD in the production of the alginate sugar nucleotide building block, GDP-ManA 2, from GDP-Man 1, including a proposed mechanism of oxidation; R = H or Ac. (B) Recent examples of sugar nucleotide-based probes and inhibitors of GMD (**3** & **4**) and a non-specific small molecule inhibitor, penicillic acid (5).

Sufferers of the autosomal recessive genetic disorder cystic fibrosis (CF) are at extremely high risk for contracting chronic lung infections. Over the lifetime of a CF patient one bacterial strain in particular, *Pseudomonas aeruginosa* (PA), becomes the dominant pathogen and causes chronic respiratory infections in over 80% of CF patients.^1^ Such strains undergo positive selection for mutations that facilitate long-term survival within the lung,^2^ and over time incur loss-of-function mutations in the mucA gene that lead to a phenomenon known as mucoid conversion; by age 16 over 90% of CF patients have infections of mucoid PA.^1^ Mucoid PA copiously secretes the exopolysaccharide alginate (Fig. 1), which presents itself macroscopically as a viscous slime and, within the resultant bacterial biofilm environment, confers resistance to current antibiotic treatments.^3,4^ Ultimately, this leads to decreased respiratory function and increased mortality rates for CF sufferers. Alginate is therefore a major virulence factor for CF lung infections and contributes deleteriously to patient life expectancy.^5^ Strategies that can stop the production of alginate in mucoid PA infection are therefore of paramount importance, as they could drastically improve the lifestyle and life expectancy of CF patients suffering from chronic infection.

Biosynthesis of alginate initiates in the cytosol and is dependent upon provision of the sugar nucleotide GDP-mannuronic acid (GDP-ManA, 2, Fig. 1).^6^ Mannuronate donor 2 is biosynthesised through a series of enzymatic transformations, starting from fructose 6-phosphate and culminates at the limiting step in alginate precursor biosynthesis,^7^ the action of GDP-mannose dehydrogenase (GMD) upon GDP-mannose 1 (Fig. 1). GMD is the product of the *algD* gene and alginate production depends upon its transcriptional activation within mucoid PA strains, switching on the system to overproduce the exopolysaccharide.^8^ Since there is no corresponding enzyme in humans, specific inhibition of GMD should produce few side effects and such a strategy could be expected to supress alginate production and interfere with exopolysaccharide formation in chronic mucoid PA infections.

We recently disclosed the first targeted sugar nucleotide probes for GMD.^9–13^ Utilising a chemoenzymatic approach enabled synthetic pyranose modification of the GDP-sugar,^14–16^ and delivered a C6-Me homologue 3(which was oxidised by GMD), and amide 4 as the first inhibitor (IC_50_ = 112 μM).^10^ Despite the value in access to such probes, molecular architectures better amenable to crossing a Gram-negative cell envelope to target the GMD active site are required. In early, seminal work surrounding the structural and kinetic characterisation of GMD,^17^ Tipton and colleagues demonstrated that penicillic acid 5 (Fig. 1b), which contains a conjugated Michael acceptor, was an irreversible, nonspecific inactivator of GMD *in vitro*, with low selectivity for the active site of the protein.^18^ In addition, the movement of the GDP-mannose binding site loop was demonstrated to be partially rate-limiting, with the interaction of Cys213-Asn252 holding the loop in a closed position during catalysis. Mutation of Cys213 to Ala213 to allow free movement of the loop was demonstrated to result in a 1.8-fold increase in *V*_max_. Considering this and with a view to moving towards a drug-like pharmacophore space for GMD inhibition, we here perform virtual screening to identify ligands for the GMD sugar nucleotide binding site, alongside potential to interact with the active site cysteine residue. Hits from this screening are verified experimentally using our previously established assay for GMD inhibition.^10,12,13^

## Results and discussion

To provide a ligand library, a unique in-house collection of 1447 natural products, and their derivatives, from the Siberian taiga and tundra was used for virtual screening with the Glide, FRED and GOLD docking algorithms.^19–24^ The library is within the parameters of Known Drug Space,^19^*i.e.*, larger than drug-like chemical space (see, Section S1 and Table S5, ESI†). For the screening using Glide and FRED, the ligands with favourable docking scores (<−9.0 kcal mol^−1^ from GLIDE and <−12.5 kcal mol^−1^ from FRED) were visually evaluated for interactions with Cys268, a water molecule and residues interacting with 2; those which interacted with less than two of these residues were discarded. For GOLD, ligands which were predicted to form multiple hydrogen bonds with key residues known to form hydrogen bonds with the co-crystallised ligand (2) were prioritised, as well as ligands with favourable scores. The results of this screening identified a combined panel of 40 compounds exhibiting favourable binding modes and scores (see ESI,† Section S1 for Glide, FRED and GOLD results, respectively).

**Fig. 2:**
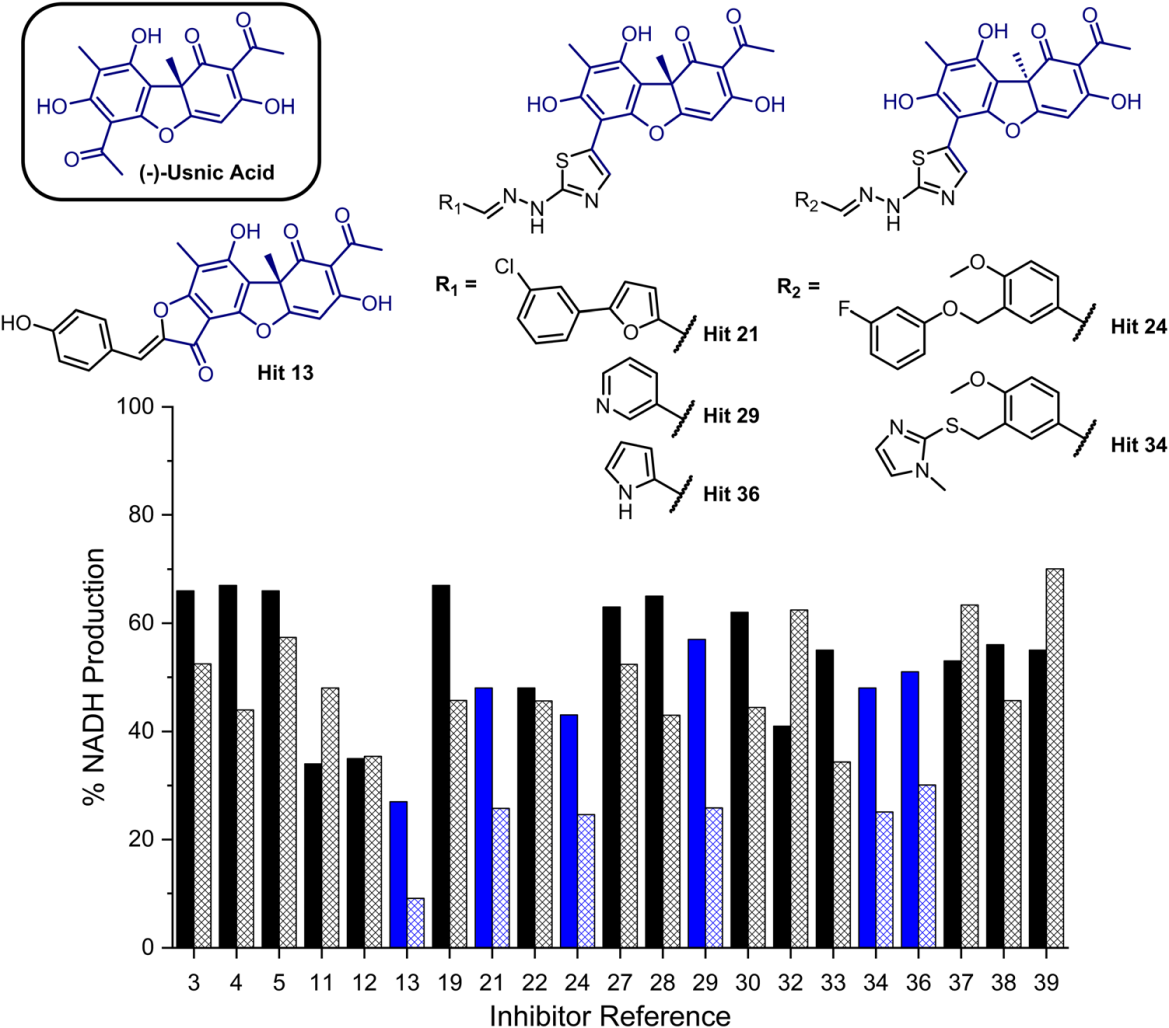
Bar chart comparing percentage NADH production in the presence of each of 21 potential inhibitors without preincubation with GMD (solid bars) and with preincubation for 1 hour with GMD (hashed bars). Complete structure panel is shown in the ESI,† Section S1.2. The 6 best performing compounds are highlighted blue. Percentage NADH production was determined relative to a positive control containing no inhibitor and 1.

We next evaluated these 40 compounds with recombinant GMD, monitoring disruption to the production of NADH in the presence of 1. We first completed this assay without any preincubation of the ligands (see ESI,† Fig. S2) and molecules where more than 70% of NADH production still occurred were discarded. Subsequently, the GMD assay was repeated with a 60 minute preincubation period for 21 compounds. From this second assay, 6 compounds reduced the production of NADH in a time-dependent manner and to below 30% of normal function (highlighted blue, Fig. 2). Consideration of the structures of these compounds revealed a common molecular scaffold, derived from usnic acid (illustrated in Fig. 2). Usnic acid is a secondary metabolite produced by lichens and has shown promising biological activity (alongside synthetic derivatives thereof),^25^ including against SARS-CoV-2.^26^

Given the similarity in activity of these hits (13, 21, 24, 29, 34 and 36, Fig. 2), we decided to focus our efforts in further investigating the strongest hit 13. This compound was found to reduce NADH production by over 90% when incubated with GMD at a concentration of 50 μM. It was thus evaluated further *in vitro*, first determining its IC_50_ and then through mass spectrometry in coordination with virtual covalent docking.

**Fig. 3:**
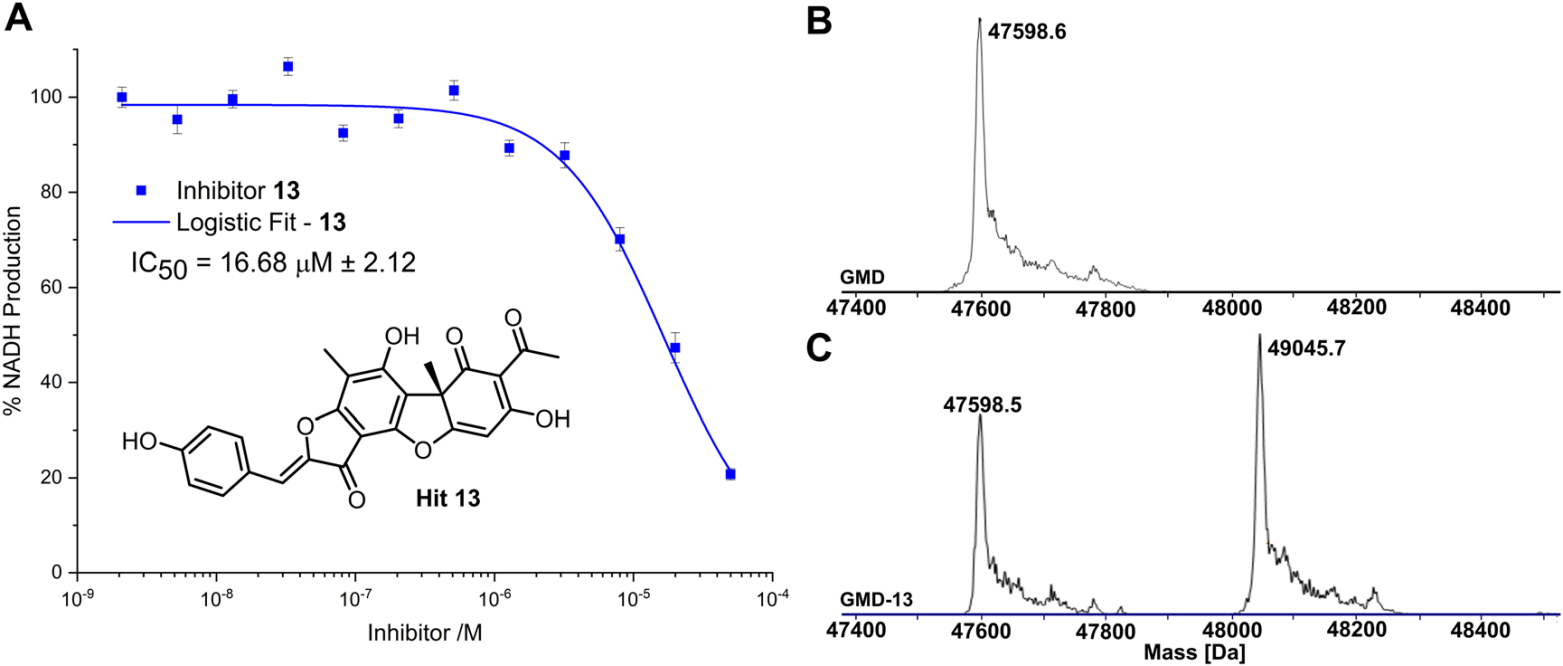
(A) Inhibition of GMD with hit 13, determined by fluorescence of NADH. Error bars indicate the standard error of three measurements. (B) ESI-MS of GMD (47598.6 Da) before incubation with 13. (C) ESI-MS of GMD after overnight incubation with 13, showing the formation of a single covalent GMD-13 adduct (49045.7 Da).

Compared to the other five time-dependent hits, compound 13 reduced NADH production to under 10% over the first 60 minutes of enzyme activity, following a 1 hour preincubation with GMD. To determine *in vitro* activity of 13, a dilution series was completed (Fig. 3A) and the IC_50_ was calculated to be 16.68 ± 2.12 μM; approximately a six-fold improvement in potency compared to our previously established C6-amide inhibitor 4.^10^ Following overnight incubation of GMD with ligand 13, ESI-MS indicated clear evidence of a single GMD-13 adduct (Fig. 3B and C). The observed [M] peak following deconvolution for the control GMD sample was 47598.6 Da (expected 47598.55 Da). For the sample incubated with inhibitor 13 (mass 446.1002 Da), two major peaks were identified at 47598.5 and 49045.7 Da, corresponding to unmodified GMD and a single GMD-13 adduct (expected 49044.65 Da), respectively.

**Fig. 4:**
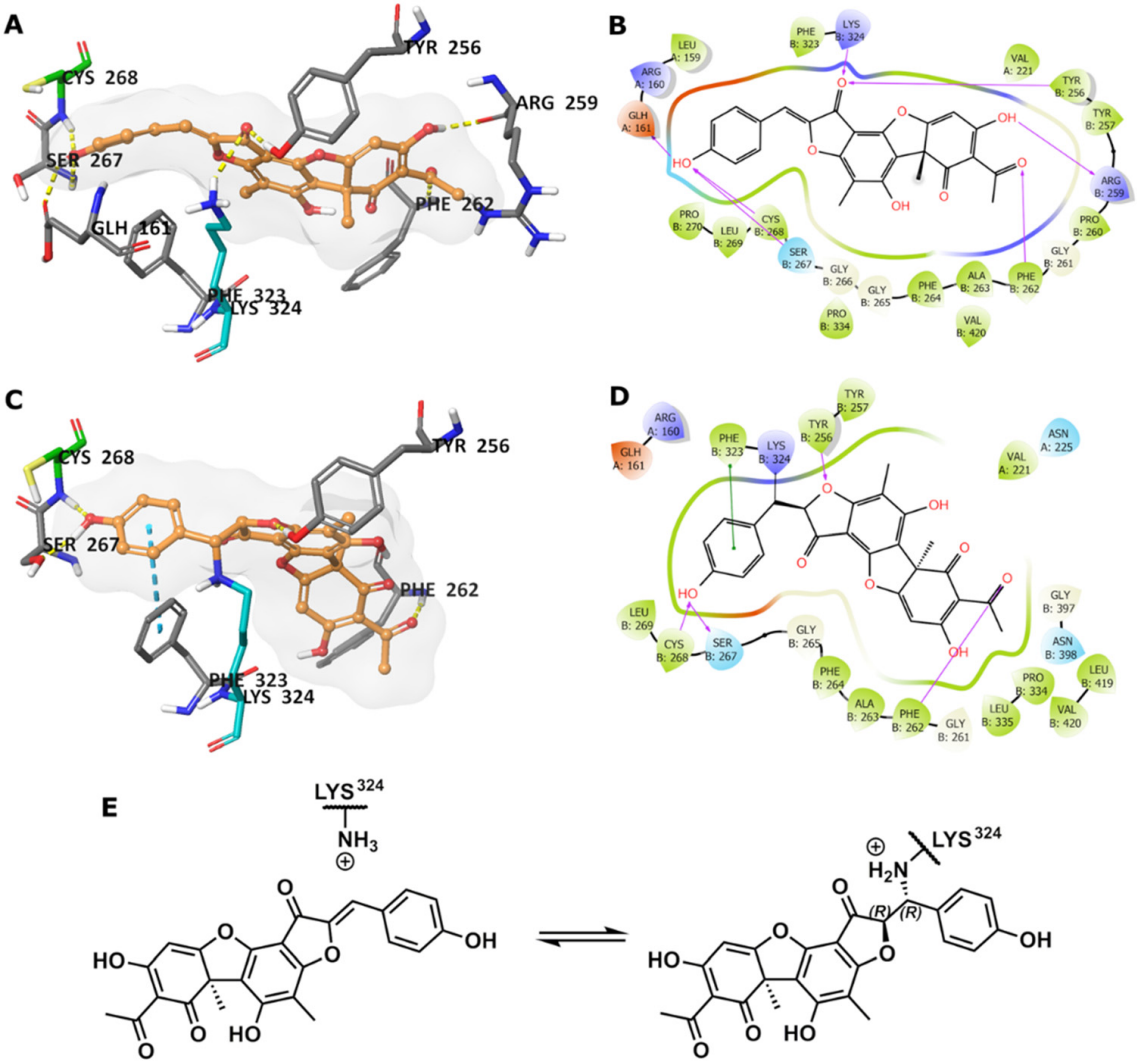
(A) Predicted binding of 13 in GMD active site. (B) The binding interactions in a 2D diagram. (C) Predicted GMD-13 adduct *via* Lys324. (D) The binding interactions in 2D diagram. (E) Indicative Lys modification of inhibitor 13*via* an aza-Michael addition. In 3D images, 13 is shown in orange stick-ball representation with molecular surface rendered, amino acid residues in grey sticks, except Cys268 in green and Lys324 in teal, and binding interactions as colour dashed lines.

To predict the binding of 13 with GMD, our docking using Glide was further optimised by applying MM-GBSA calculations, allowing flexibility to nearby residues. The free binding energy of 13 was calculated as −56.9 kcal mol^−1^ for the pose stabilised through electrostatic interactions with key residues, including Glu161, Tyr256, Arg259, Phe261, Cys268, and Lys324 (Fig. 4A and B). Whilst this predicted binding of 13 did not suggest a likely covalent engagement with Cys268, it did with Lys324 whose side chain was positioned close to a Michael acceptor located between a phenolic end group and a fused ketofuran in 13. Albeit still uncommon for lysine (relative to cysteine), there are emergent examples of aza-Michael reactions on native proteins.^27,28^

Covalent docking, followed by an MM-GBSA calculation,^29^ was performed to test the possibility and favourability of a GMD-13 adduct *via* Lys324 (highlighted as teal coloured adduct in Fig. 4C). This predicted covalent complex proved thermodynamically favourable with an enhanced free binding energy of −68.2 kcal mol^−1^ compared to the non-bonded complex, as the electrostatic interactions with the key residues, including Cys268, were mostly retained (Fig. 4C and D). This modelling study predicts a Michael addition of Lys324 in GMD to 13 (Fig. 4E), resulting in a covalent adduct and supports our observed ESI-MS data. Furthermore, the non-covalent docking indicates the carbonyl of the furanone within inhibitor 13 may interact with Tyr256 (Fig. 4B). If occurring, this could provide Lewis acidity and lower the energy of the C=C π* orbital, favouring addition of lysine at this position.

## Conclusion

Using virtual screening, a panel of 1447 compounds were evaluated as potential ligands for the *Pseusomonas aeruginosa* GDP-mannose dehydrogenase (GMD) sugar nucleotide binding site. This identified a panel of 40 potential hits, narrowed to 21 active compounds using an NADH reporter assay with recombinant GMD *in vitro*. Furthermore, this panel was refined to 6 compounds observed to reduce NADH production by over 70% in a time-dependent manner following ligand–enzyme preincubation. Investigation of the structure of these 6 compounds revealed a common drug-like pharmacophore closely related to the natural product usnic acid. Of these time-dependent hits, one molecule (13) was determined to have an IC_50_ of 16.7 μM. Further validation by mass spectrometry revealed only a single adduct was formed when incubated with GMD and covalent docking suggests Lys324 as the possible alkylation site.

Taken together, these results not only identify the first small molecule inhibitor of GMD, but also underpin necessity for further exploration of this natural product to confirm its mechanism of action and wider optimisation of its inhibitory activity (from a medicinal chemistry perspective). Furthermore, this GMD conjugable natural product scaffold opens a doorway to explore the development of chemical biology tools (*e.g.*, tunable fluorescent probes) to detect this important bacterial protein.

## Data availability statement

The data that support the findings of this study are available from the corresponding author upon reasonable request.

## Author contributions

CRediT: Jonathan P. Dolan methodology, investigation, formal analysis, data curation, visualisation, writing – original draft, writing – review & editing; Sanaz Ahmadipour methodology, investigation, formal analysis, data curation; Alice J. C. Wahart investigation, formal analysis, visualisation; Aisling Ní Cheallaigh resources, supervision; Suat Sari methodology, investigation, formal analysis, data curation, visualisation, writing – original draft, funding acquisition; Chatchakorn Eurtivong investigation, formal analysis, data curation; Marcelo A. Lima resources, supervision; Mark A. Skidmore resources, supervision; Konstantin P. Volcho resources; Jóhannes Reynisson conceptualization, methodology, investigation, formal analysis, data curation, supervision, project administration funding acquisition; Robert A. Field conceptualization, methodology, writing – review & editing, supervision, project administration, funding acquisition; Gavin J. Miller conceptualization, methodology, writing – original draft, writing – review & editing, visualisation, supervision, project administration, funding acquisition.

## Conflicts of interest

There are no conflicts to declare.

## Supplementary Material

CB-004-D3CB00126A-s001
