# Correction: Virtual screening, identification and *in vitro* validation of small molecule GDP-mannose dehydrogenase inhibitors

**DOI:** 10.1039/d4cb90026j

**Published:** 2024-07-08

**Authors:** Jonathan P. Dolan, Sanaz Ahmadipour, Alice J. C. Wahart, Aisling Ní Cheallaigh, Suat Sari, Chatchakorn Eurtivong, Marcelo A. Lima, Mark A. Skidmore, Konstantin P. Volcho, Jóhannes Reynisson, Robert A. Field, Gavin J. Miller

**Affiliations:** a Lennard-Jones Laboratory, School of Chemical & Physical Sciences, Keele University Keele Staffordshire ST5 5BG UK g.j.miller@keele.ac.uk; b Centre for Glycoscience, Keele University Keele Staffordshire ST5 5BG UK; c Hornbeam Building, School of Pharmacy & Bioengineering, Keele University Keele Staffordshire ST5 5BG UK; d School of Life Sciences, Keele University Keele Staffordshire ST5 5BG UK; e Department of Chemistry & Manchester Institute of Biotechnology, The University of Manchester 131 Princess Street Manchester M1 7DN UK; f Hacettepe University, Faculty of Pharmacy, Department of Pharmaceutical Chemistry 06100 Ankara Turkey; g Department of Pharmaceutical Chemistry, Faculty of Pharmacy, Mahidol University 447 Si Ayutthaya Road, Ratchathewi Bangkok 10400 Thailand; h N. Vorozhtsov Novosibirsk Institute of Organic Chemistry, Siberian Branch of the Russian Academy of Sciences 630090 Novosibirsk Russia

## Abstract

Correction for ‘Virtual screening, identification and *in vitro* validation of small molecule GDP-mannose dehydrogenase inhibitors’ by Jonathan P. Dolan *et al.*, *RSC Chem. Biol.*, 2023, **4**, 865–870, https://doi.org/10.1039/D3CB00126A.

The authors regret an error in the published article whereby the stereochemistry of one carbon within compound **13** in Fig. 2 and 3 was incorrect. All other designations of compound **13** in the article and supplementary information were correct. The corrected article figures are below.

**Fig. 2:**
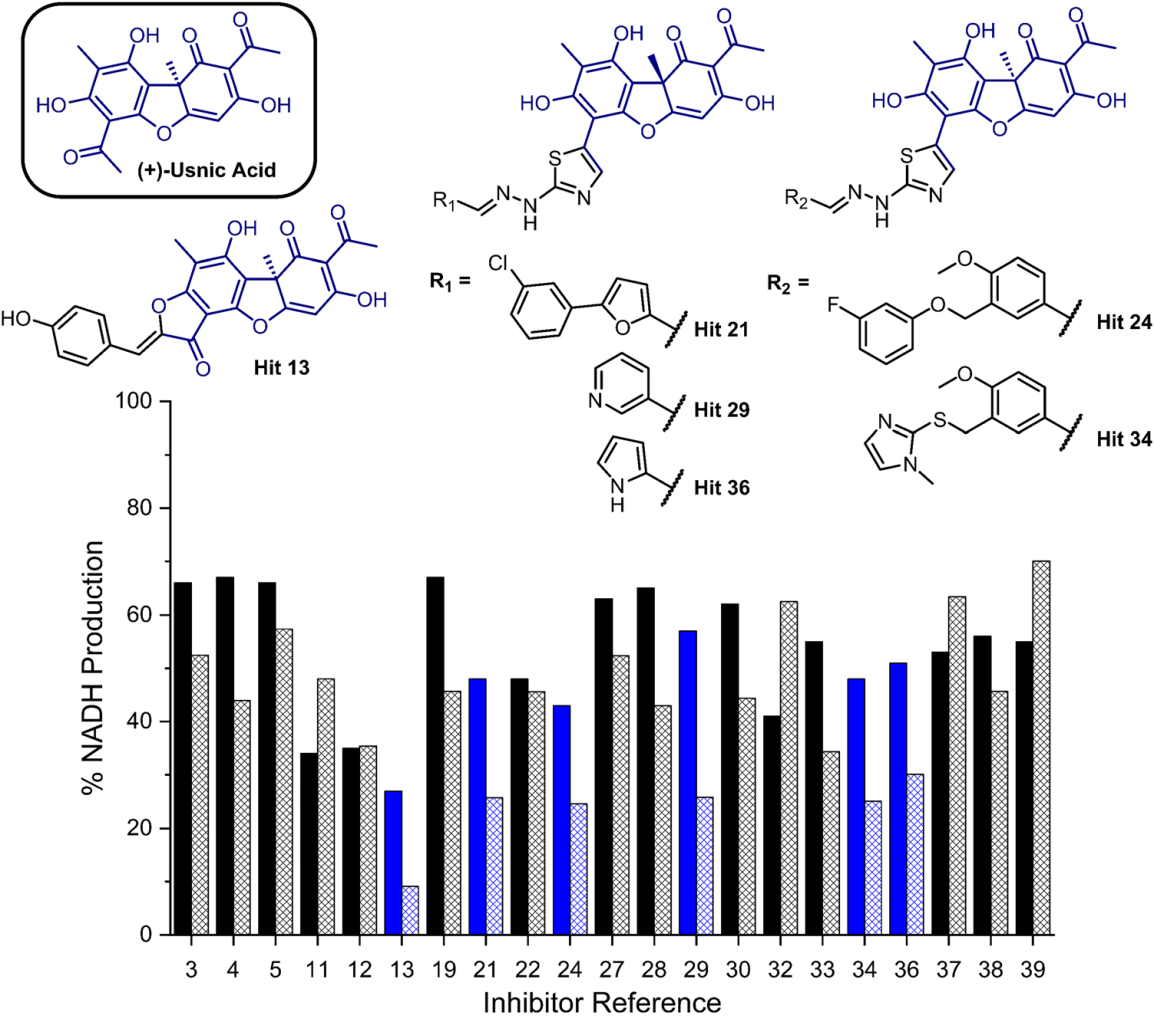
Bar chart comparing percentage NADH production in the presence of each of 21 potential inhibitors without preincubation with GMD (solid bars) and with preincubation for 1 hour with GMD (hashed bars). Complete structure panel is shown in the ESI,† Section S1.2. The 6 best performing compounds are highlighted blue. Percentage NADH production was determined relative to a positive control containing no inhibitor and **1**.

**Fig. 3:**
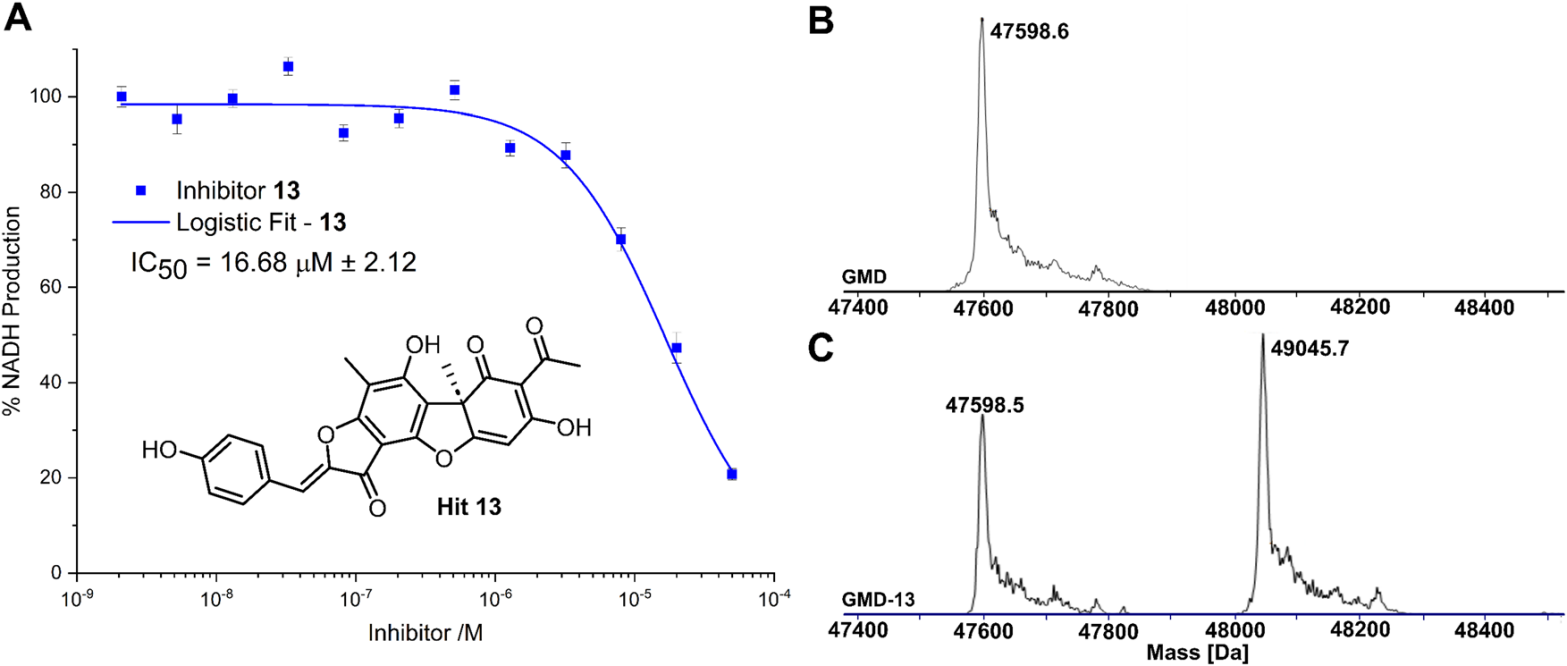
(A) Inhibition of GMD with hit **13**, determined by fluorescence of NADH. Error bars indicate the standard error of three measurements. (B) ESI-MS of GMD (47598.6 Da) before incubation with **13**. (C) ESI-MS of GMD after overnight incubation with **13**, showing the formation of a single covalent GMD-**13** adduct (49045.7 Da).

The Royal Society of Chemistry apologises for these errors and any consequent inconvenience to authors and readers.

