# Supplementary material for: Virtual screening, identification and in vitro validation of small molecule GDP-mannose dehydrogenase inhibitors
Source: RSC Chem Biol. 2023 Aug 29;4(11):865–70. doi: 10.1039/d3cb00126a (PMC10619135; doi:10.1039/d3cb00126a)
Supplement: CB-004-D3CB00126A-s001 [file CB-004-D3CB00126A-s001.pdf]

Electronic Supplementary Material (ESI) for RSC Chemical Biology.
This journal is © The Royal Society of Chemistry 2023

Experimental and SI

Virtual screening, identification and *in vitro* validation of small molecule GDP-mannose dehydrogenase inhibitors

Jonathan P. Dolan,[1,2] Sanaz Ahmadipour,[5] Alice J. C. Wahart,[1,2] Aisling Ní Cheallaigh,[1,2] Suat Sari,[6] Chatchakorn Eurtivong,[7] Marcelo A. Lima,[2,4] Mark A. Skidmore,[2,4] Konstantin P. Volcho,[8] Jóhannes Reynisson,[2,3] Robert A. Field[5] and Gavin J. Miller[1,2]*

[1]Lennard-Jones Laboratory, School of Chemical & Physical Sciences, Keele University, Keele, Staffordshire, ST5 5BG, UK.
[2]Centre for Glycoscience, Keele University, Keele, Staffordshire, ST5 5BG, UK.
[3]Hornbeam Building, School of Pharmacy & Bioengineering, Keele University, Keele, Staffordshire, ST5 5BG, UK.
[4]School of Life Sciences, Keele University, Keele, Staffordshire, ST5 5BG, UK.
[5]Department of Chemistry & Manchester Institute of Biotechnology, The University of Manchester, 131 Princess Street, Manchester, M1 7DN, UK
[6]Hacettepe University, Faculty of Pharmacy, Department of Pharmaceutical Chemistry, 06100, Ankara, Turkey.
[7] Department of Pharmaceutical Chemistry, Faculty of Pharmacy, Mahidol University, 447 Si Ayutthaya Road, Ratchathewi, Bangkok 10400, Thailand.
[8]N. Vorozhtsov Novosibirsk Institute of Organic Chemistry, Siberian Branch of the Russian Academy of Sciences, 630090 Novosibirsk, Russia.

*Author for correspondence: g.j.miller@keele.ac.uk

Section 1: Virtual screening using Glide, FRED and GOLD

Virtual screening methodology using Glide and FRED

The Known Drug Space (KDS)-focused[1] Siberian in-house library of 1447 compounds, which was optimized previously, was used for docking on Glide (2019-4, Schrödinger LLC, New York, NY)[2] and FRED (v3.3.1.2: Open Eye Scientific Software; Santa Fe, NM).[3] Crystal structure of GMD from *P. aeruginosa* (PDB ID: 1MV8[4]) was downloaded from the RCSB Protein Data Bank (www.rcsb.org)[5] and prepared for Glide using Protein Preparation Wizard of Maestro (MacroModel: 2019-4, Schrödinger LLC, New York, NY), where unwanted residues were removed, bond orders, explicit hydrogens, partial charges (OPLS3e) were assigned, ionization states and H bonds were set[6,7]. Make Receptor (v3.3.1.2: Open Eye Scientific Software; Santa Fe, NM) was used to prepare the protein for FRED docking. Grid maps of the GMD active site (centroid: -42.68, 6.62, 21.86; volume: 27,000 Å^3) was generated using Receptor Grid Generator panel of Maestro for Glide and Make Receptor (v3.3.1.2: Open Eye Scientific Software; Santa Fe, NM) for FRED. The ligands were docked to the active site using Glide (2019-4, Schrödinger LLC, New York, NY) at extra precision (XP) mode and FRED (v3.3.1.2: Open Eye Scientific Software; Santa Fe, NM) using high resolution mode with 50 runs per ligand. The results were ranked by XP GScore from Glide and FRED Chemgauss4 score from FRED and visually evaluated. The docking score of the best pose of each ligand was noted accordingly.

Virtual screening methodology using GOLD

The GOLD[8,9] version 5.6.2 software suite was also used as the docking engine to predict for biologically active hits from the same compound library. The same protein set up and centre of binding coordinates were used with 10 Å radius. The basic amino acids lysine and arginine were defined as protonated. Furthermore, aspartic and glutamic acids were assumed to be deprotonated. Three scoring functions were implemented to validate for the predicted binding modes: Goldscore (GS),[10] Chemscore (CS),[11] Chem Piecewise Linear Potential (ChemPLP),[12] and Astex Statistical Potential (ASP).[13]

Covalent docking and MM-GBSA calculations

Covalent docking was preformed using CovDock[14] panel of Maestro (2019-4, Schrödinger LLC, New York, NY) with the same centroid coordinates above. For modelling of the Michael addition of lysine, a custom cdock file was generated manually. A maximum 200 initial poses were kept for refinement and 50 final poses were generated. MM-GBSA was performed using Prime panel of Maestro (2019-4, Schrödinger LLC, New York, NY) with VSGB solvation model according to the OPLS3e force field parameters. Residues at 4 Å away from **13** were kept flexible and the optimized complexes were sampled using force field minimization.

1.1: Glide & FRED

Virtual Screening results using Glide and FRED: The results from Glide and FRED are listed according to the docking scores, the ligands with docking score < -9.0 kcal/mol from Glide (top 12 compounds) and < -12.5 kcal/mol from FRED (top 20 compounds) were visually evaluated regarding integrations with the Cys268, the water molecule, and the residues interacting with GDP-mannuronic acid and those interacting with less than two of these residues were discarded.

Table S1. Top-scoring compounds according to Glide, their scores and the key GMD residues in electrostatic interactions (H bond, π-π, π-cation, salt bridge, halogen bond).

Compound	Score (kcal/mol)	Residues
AF-122	-11.2	Arg259B, Gly265B, Phe323B, Lys324B, Ala325B, Gly399B
OL9-162-2	-10.7	Phe262B, Gly265B, Lys324B
AF-234	-10.3	**Cys268B**, Lys324B, Leu419B
AF-195	-10.2	Glu161A, Arg255B, Tyr256B, Arg259B, Phe323B, Ala325B, Gly399B
AF-28	-10.1	Gly265B, Leu269B, Gly397B, Gly399B
UDCA	-9.7	Phe262B, Gly265B, **Cys268B**, Leu269B, Phe323B
AF-37	-9.7	Tyr256B, Gly265B, Lys324B, Gly397B, Gly399B
AF-183	-9.6	Phe262B, Gly265B, Phe323B, Lys324B
nli-8	-9.6	Tyr257B, Phe262B, Gly265B, Lys324B
DS-411	-9.5	Tyr256B, Gly265B, Gly397B
OL9-187-2	-9.3	Gly265B, Lys324B
AF-125	-9.0	Lys210A, Asn214A, Tyr257B, Gly265B, Phe323B

Table S2. Top-scoring compounds according to FRED, their scores and the key GMD residues in electrostatic interactions (H bond, π-π, π-cation, salt bridge, halogen bond).

Compound	Score (kcal/mol)	Residues
GA	-13.4	Arg259B, Gly265B, Ser267B, **Cys268B**, Leu269B

OL6-123	-13.2	Glu157A, Lys210A, Asn214A, Tyr256B, Tyr257B, Ala263B, Gly397B
OL6-129	-13.1	Tyr256B, Tyr257B, Ala263B, Gly265B
OL6-127	-13.1	Glu157A, Lys210A, Asn214A, Tyr256B, Tyr257B, Ala263B, Gly397B
AF-160	-13.0	Arg259B, Gly265B, Phe323B, Gly397B
DCA-Ep-3.1	-13.0	Glu161A, Tyr256B
OL8-122	-12.9	Leu159A, Glu161A, Tyr256B, Ala263B, Lys324B, Gly397B
DS-342-1	-12.8	Glu161A, Tyr256B, Tyr257B, Phe262B, Gly265B, Ser267B, Lys324B
OL7-135	-12.8	Tyr256B, Tyr257B, Ala263B, Gly397B
CDCA	-12.8	Arg259B, Ala263B, Ser267B, **Cys268B**, Leu269B
nli-4	-12.8	Tyr257B, Arg259B, Lys324B
nli-6	-12.7	Tyr257B, Gly265B, Leu269
maa-134	-12.7	Tyr256B, Tyr257B, Lys324B
TX-13	-12.6	Tyr257B, **Cys268B**, Leu269, Phe323B
DS-351	-12.6	Tyr256B, Ala263B, Gly265B, Lys324B, Gly397B
I9-33-1	-12.6	Tyr256B, Tyr257B, Lys324B
DS-465-1	-12.5	Leu159A, Tyr256B, Phe262B, Gly265B
OL9-62	-12.5	Leu159A, Glu161A, Tyr256B, Gly265B, Ser267B, Phe323B
CA-Me	-12.5	Tyr256B, Phe262B, Val420B
DCA-Ep-2.1	-12.5	Tyr256B, **Cys268B**, Leu269

Virtual screening results using GOLD

A 3D library of 1447 small molecules from the in-house library were docked into the catalytic site and virtually screened (see methodology) at 30% efficiency at 20 GA runs. Efficiency indicates the thoroughness in which the algorithm searches for a thermodynamically favourable binding pose in the enzyme active site. All compounds with a hydrogen bonding score of less than 1.0 were removed and the top scoring 200 compounds from each of GS, CS and ChemPLP scoring functions were kept resulting in 263 compounds. The resulting 263 compounds were virtually screened using the same protocol at 100% efficiency at 50 GA runs. All compounds with hydrogen bonding scores of less than 1.0 were removed and the top scoring 100 compounds from each of the three scoring functions were selected for visual inspection leaving a pool of 179 compounds. Most of the compounds at this stage formed hydrogen bond interactions with some of the residues involved in stabilising the co-crystallised GDP-mannuronic acid. Considering numerous hydrogen bonding residues were formed with the co-crystallised ligand, the hydrogen bond scores and networks were prioritised, *i.e.*, compounds predicted to form numerous hydrogen bonds with important residues. A total of 8 compounds were chosen (Table **3**).

Table S3. The docking scores and hydrogen bonding residues of the co-crystallised ligand and the 8 predicted inhibitors.

**Compound**	**GS**	**GS hb**	**CS**	**CS hb**	**ChemPLP**	**ChemPLP hb**	**Hydrogen bonding residues**

**Co-crystallised ligand**	110.55	35.23	22.90	11.66	108.42	15.84	Phe158, Leu159, Glu161, Lys210, Asn214, Tyr256, Tyr257, Arg259, Phe262, **Cys268**, Lys324
**BA-AM1-41**	44.96	8.17	45.14	4.48	81.24	4.75	Lys210, Asn214, Glu157m water, Leu159, Lys324
**PI-389**	68.56	7.64	39.34	2.68	85.03	2.54	Glu157, Lys324, Arg259
**BA-AM1-46**	50.75	6.6	50.73	4.75	95.53	5.85	Lys324, Lys210, Asn214, water, Glu157
**AF-80**	59.02	4.39	34.58	2.27	85.51	2.09	Phe262, Tyr257, Tyr256, Lys210
**OL7-27**	56.12	4.85	35.24	6.13	80.57	8.56	Phe323, Gly265, Tyr257, Asn214, Lys210, Glu157, water
**BA-AM1-40**	41.72	7.03	44.29	4.51	85.82	5.53	Leu159, Lys324, Glu157, Lys210, Asn214, water
**AF-101**	68.9	4.65	32.74	1.99	69.43	3.9	Asn214, Tyr256, Tyr257, Ser333, Arg259
**OL8-118**	69.93	4.71	32.82	5.41	79.35	4.66	**Cys268**, Leu269, Tyr256, Arg259

1.2: Structures of Virtual Screening Hits

Table S4. The structure of virtual screening hits and LogP values.

Inhibitor Number	Library Reference	LogP	Structure
**1**	GA	5.141	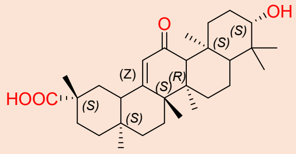 Molecular Weight: 470.6940
**2**	PI-389	4.914	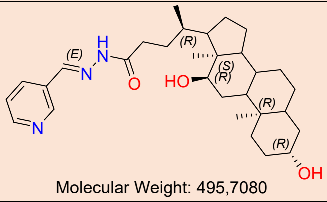 Molecular Weight: 495,7080
**3**	DCA-Ep3.1	4.327	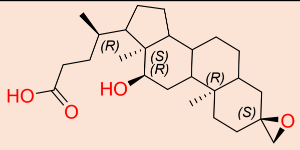 Molecular Weight: 404,5910
**4**	OL7-135	2.67	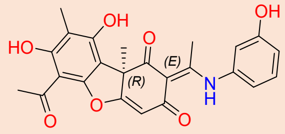 Molecular Weight: 435,4320
**5**	OL6-123	3.938	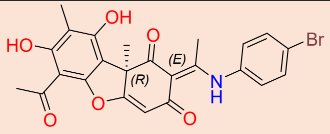 Molecular Weight: 498,3290

**6**	OL6-127	3.866	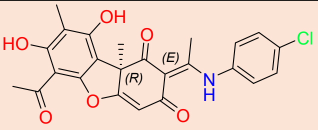<br>Molecular Weight: 453,8750
**7**	CDCA	3.772	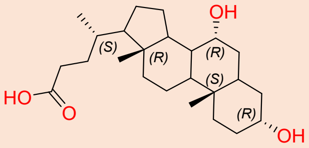<br>Molecular Weight: 392,5800
**8**	AF-195	4.091	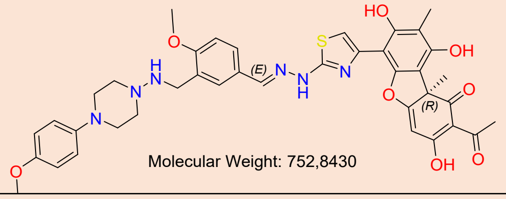<br>Molecular Weight: 752,8430
**9**	nli-4	4.303	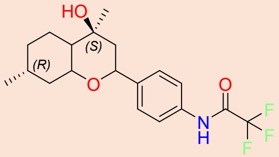<br>Molecular Weight: 371,4002
**10**	OL7-27	1.792	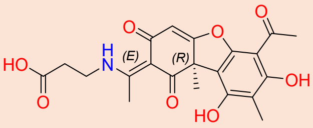<br>Molecular Weight: 415,3980
**11**	OL9-162	5.703	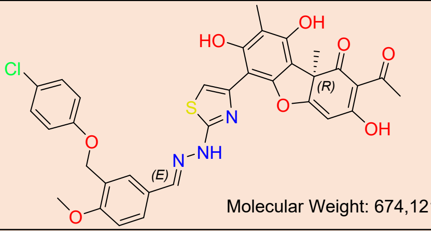<br>Molecular Weight: 674,1210
**12**	AF-122	5.477	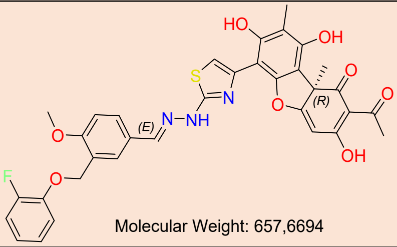<br>Molecular Weight: 657,6694
**13**	DS-465-1	2.352	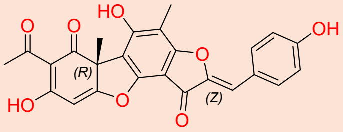<br>Molecular Weight: 446,4110
**14**	DS-342-1	2.388	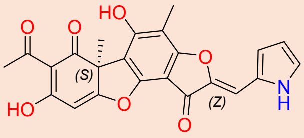<br>Molecular Weight: 419,3890

**15**	OL6-129	3.605	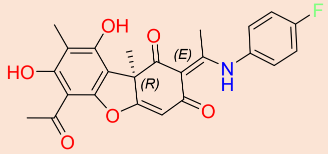 Molecular Weight: 437,4234
**16**	DCA-Ep2.1	4.215	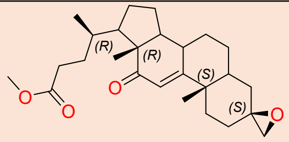 Molecular Weight: 414,5860
**17**	TX-13	4.942	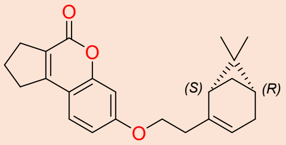 Molecular Weight: 350,4580
**18**	nli-2	5.749	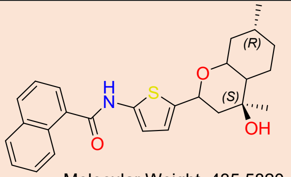 Molecular Weight: 435,5820
**19**	I9-33 (I9-33-1)	4.231	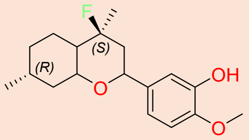 Molecular Weight: 308,3934
**20**	BA-AM1-41	5.47	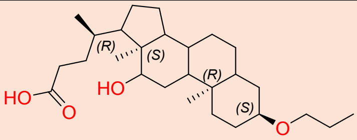 Molecular Weight: 434,6610
**21**	AF-234	4.791	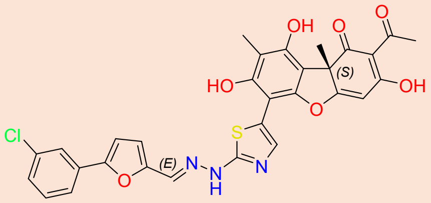 Molecular Weight: 604,0300
**22**	AF-183	5.465	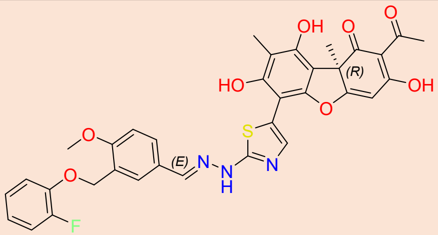 Molecular Weight: 657,6694
**23**	nli-6	5.836	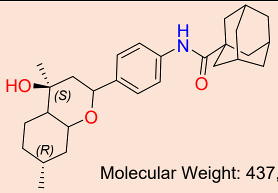 Molecular Weight: 437,6240

**24**	AF-80	5.466	Molecular Weight: 657,6694 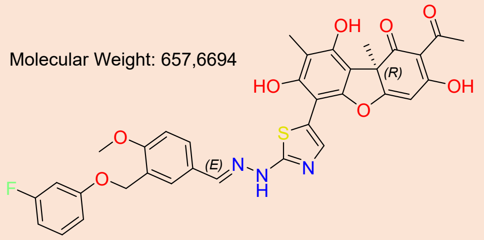
**25**	OL9-62	3.81	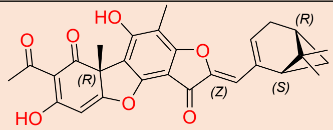 Molecular Weight: 474,5090
**26**	AF-101	3.456	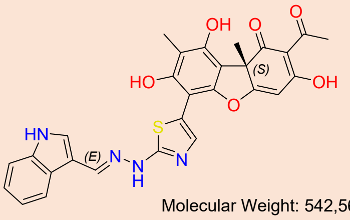 Molecular Weight: 542,5660
**27**	BA-AM1.40	5.674	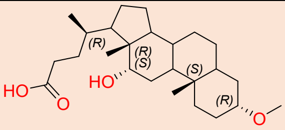 Molecular Weight: 406,6070
**28**	UDCA	3.757	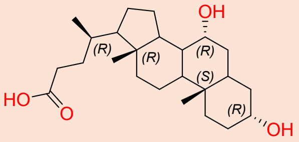 Molecular Weight: 392,5800
**29**	AF-28	2.441	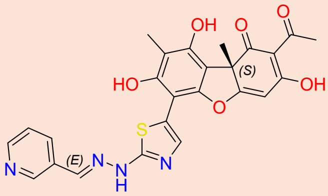 Molecular Weight: 504,5170
**30**	OL8-118	2.874	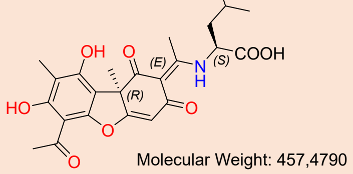 Molecular Weight: 457,4790
**31**	maa-134	4.703	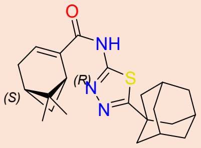 Molecular Weight: 383,5540

**32**	AF-125	5.919	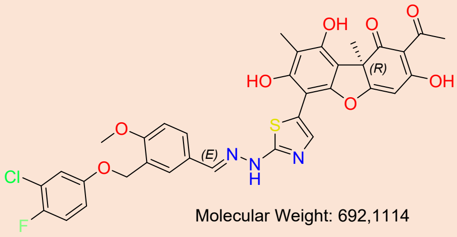 Molecular Weight: 692,1114
**33**	AF-160	3.505	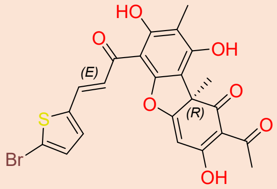 Molecular Weight: 517,3460
**34**	OL9-187	4.682	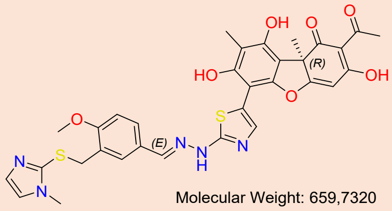 Molecular Weight: 659,7320
**35**	CA-Me	3.33	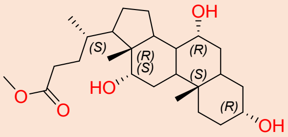 Molecular Weight: 422,6060
**36**	AF-37	2.644	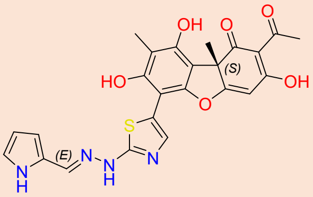 Molecular Weight: 492,5060
**37**	OL8-122	2.118	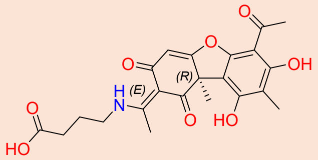 Molecular Weight: 429,4250
**38**	DS-351	4.52	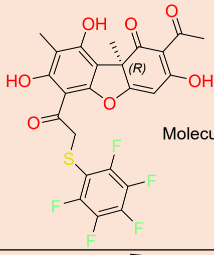 Molecular Weight: 542,4290
**39**	BA-AM1-46	6.483	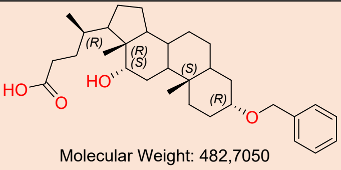 Molecular Weight: 482,7050

**40**	DS-411	2.08	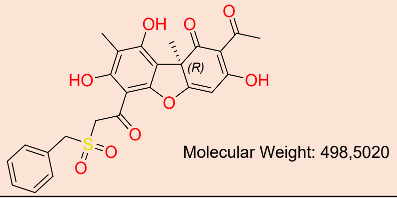

Table S5. Definition of lead-like, drug-like and Known Drug Space (KDS) in terms of molecular descriptors. The values given are the maxima for each descriptor for the volumes of chemical space used (Zhu, F.; Logan, G.; Reynisson, J. Wine Compounds as a Source for HTS Screening Collections. A Feasibility Study. Mol.Inf. **2012**, *31*, 847 – 855.).

	**Lead-like Space**	**Drug-like Space**	**Known Drug Space**
Molecular weight (g mol^{-1})	300	500	800
Lipophilicity (Log P)	3	5	6.5
Hydrogen bond donors (HD)	3	5	7
Hydrogen bond acceptors (HA)	3	10	15
Polar surface area (Å^2) (PSA)	60	140	180
Rotatable bonds (RB)	3	10	17

1.3: Pymol code for GMD Visualisation

Cartoon View

```
reinitialize
fetch 1MUU, async=0
remove solvent + inorganic
remove chain A+B
select bb, name c+o+n+ca
select sidechains, !bb
select sc, polymer & !bb + name ca + PRO/N
delete sidchains
count_atoms bb
util.cbc
as cartoon, bb
color black, organic
bg_color white
orient
select cys, (r. cys)
color red, cys
show sticks, cys
set spec_reflect, off
set ray_shadows, on
set ray_trace_mode, 1
set ray_trace_color, black
set hash_max, 800

## Manually delete GLU+FRU, Color NAD Orange, Color GDX Blue ##
```

Surface View

```
reinitialize
fetch 1MUU, async=0
remove solvent + inorganic
remove chain A+B
orient
bg_color white
as cartoon, all
color gray, all
select cys, (r. cys)
show sticks, cys
color red, cys
show surface, polymer
set transparency, 0.1
show sticks, organic
color black, organic
set cartoon_smooth_loops, 0
set hash_max, 600
set spec_power=200
set spec_refl=1.5
set surface_quality, 2
set antialias, 2

## Manually delete GLU+FRU, Color NAD Orange, Color GDX Blue ##
```

1.4: Library Compounds & Purity

All compounds were used within this study were received as gifts from the lab of Konstantin P. Volcho (N.N. Vorozhtsov Novosibirsk Institute of Organic Chemistry, Russia) & used without additional purification. All the compounds tested were reported to have a purity of >98% (by HPLC).[15–20]

Following identification of compound **13** as a potential inhibitor of GMD, additional characterisation was performed by NMR to ensure purity. 1H NMR data matched the previously reported literature data.[20]

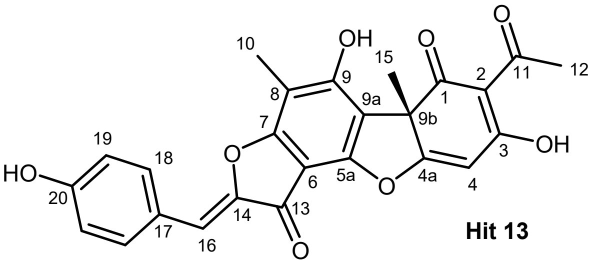

Hit 13

1H NMR (800 MHz, DMSO) δ 10.20 (1 H, s, OH-20), 7.84 (2 H, d, *J* = 8.6 Hz, H-19), 6.91 (2 H, d, *J* = 8.7 Hz, H-18), 6.79 (1 H, s, H-16), 6.29 (1 H, br.s, H-4), 2.56 (3 H, br. s, H-12), 2.25 (3 H, s, H-10), 1.75 (3 H, s, H-15).

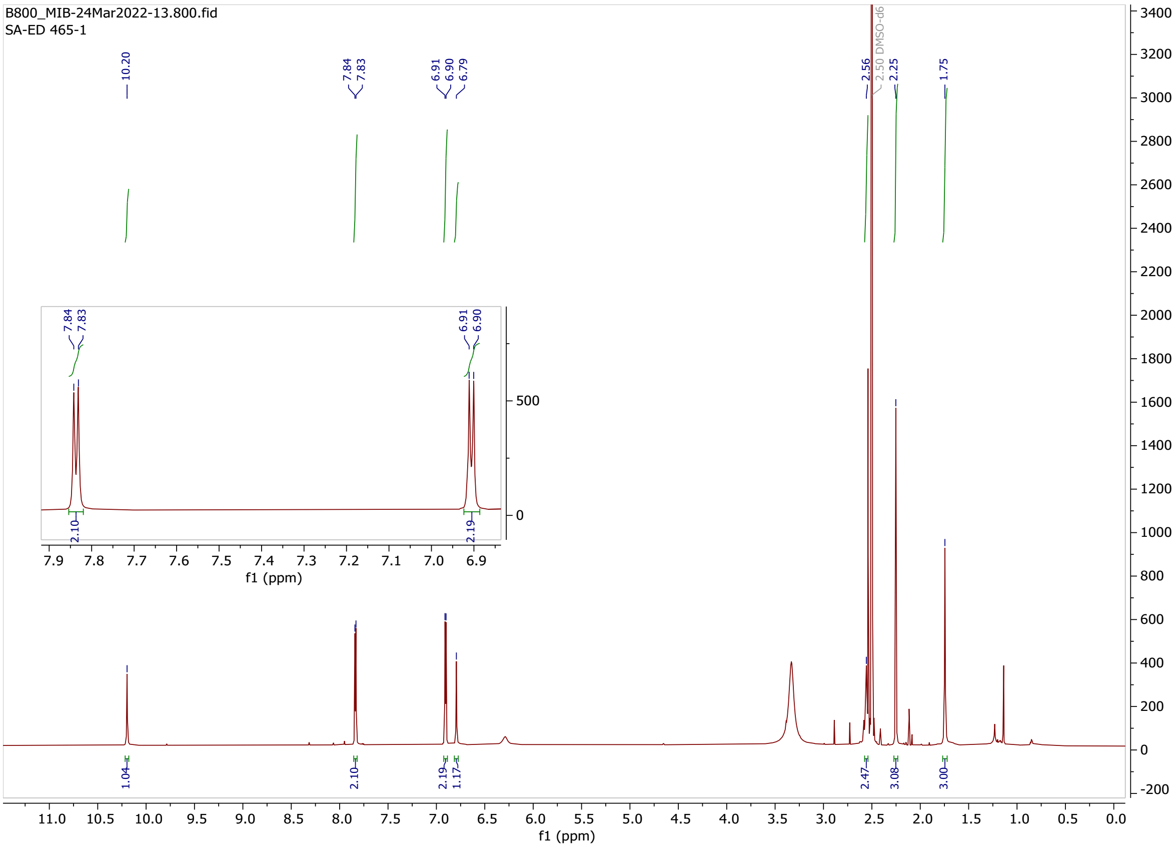

Figure S1. 1H NMR of compound **13**

Section 2: GMD expression

The recombinant plasmid (pET-3a) containing the *algD* gene encoding for GDP-mannose dehydrogenase (GMD) from P. aeruginosa was kindly donated by P. Tipton. The plasmid was transformed into *E. coli* soluBL21(DE3) chemically competent cells and the transformant grown according to the literature.[4,21]

Briefly, 1 L of the transformant in LB medium containing the appropriate antibiotic (carbenicillin, 100 µg/mL) was incubated at 37 °C with gentle shaking in baffled flasks until an OD_{600} of 0.6–0.8 was reached. Heterologous protein expression was induced by adding isopropyl β-D-1-thiogalactopyranoside (IPTG) to a final concentration of 0.4 mM, followed by incubation at 37 °C for 4 hours at 180 rpm. Afterwards the cells were harvested by centrifugation (4000 *x g*, 4 °C, 20 mins) and stored at –80 °C until use. Frozen cells were thawed in 20 mM HEPES (pH 7.5), 150 mM NaCl supplemented with DNase A (10 µg/ml, Sigma) and proteinase inhibitor cocktail (Roche), then lysed by sonication on ice. The supernatant was recovered by centrifugation (20,000 *x g*, 4 °C, 20 min) and nucleic acid precipitated through the addition of protamine sulfate (5 mg per gram wet cell pellet) and incubated on ice for 30 mins. Precipitated nucleic acid removed by centrifugation (20,000 *x g*, 4 °C, 20 min), the crude protein solution was fractionated with ammonium sulfate, with GMD precipitating between 45 and 60% saturation. Protein pellets were redissolved in 20 mM HEPES (pH 7.5), 150 mM NaCl and purified using an ÄKTA pure FPLC system (GE Healthcare) by gel filtration chromatography using a Superdex S200 16/600 column (GE Healthcare). Proteins were eluted with 20 mM HEPES (pH 7.5) and 150 mM NaCl at the flow rate of 1 ml/min. GMD comprising fractions were combined and concentrated to ~4.5 mg/mL

(concentration determined by Pierce™ BCA assay, ThermoFisher or Bradfords Assay, Sigma). Concentrated GMD was then divided into aliquots and stored at -80°C until required.

Section 3: Plate based screening of virtual hits with GMD

3.1: Initial screening of all 40 compounds with no preincubation

The assay was performed in 96-well flat bottomed, non-binding, polystyrene microtiter plates (Grenier 655906). NAD^+ (200 µM), inhibitor (50 µM) and GMD (50 µg/mL) were prepared in 50 mM sodium phosphate (pH 7.4) containing 0.5 mM $MgCl_2$ and 1 mM DTT. A solution of GDP-Man (final: 50 µM) was added to the plate and the fluorescence was measured at 25 °C for 30 minutes using a BMG labtech FLUOStar Omega microplate reader (excitation 355 nm; emission 460 nm). The limits of detection were analysed by control samples as followed: positive control contained no inhibitor; negative control contained no inhibitor or GDP-Man.

Data Processing

Initial rate of fluorescence increase was calculated over the first 20 minutes. The fluorescence was converted to % NADH production by comparison with the maximum and minimum values obtained from the positive and negative controls.

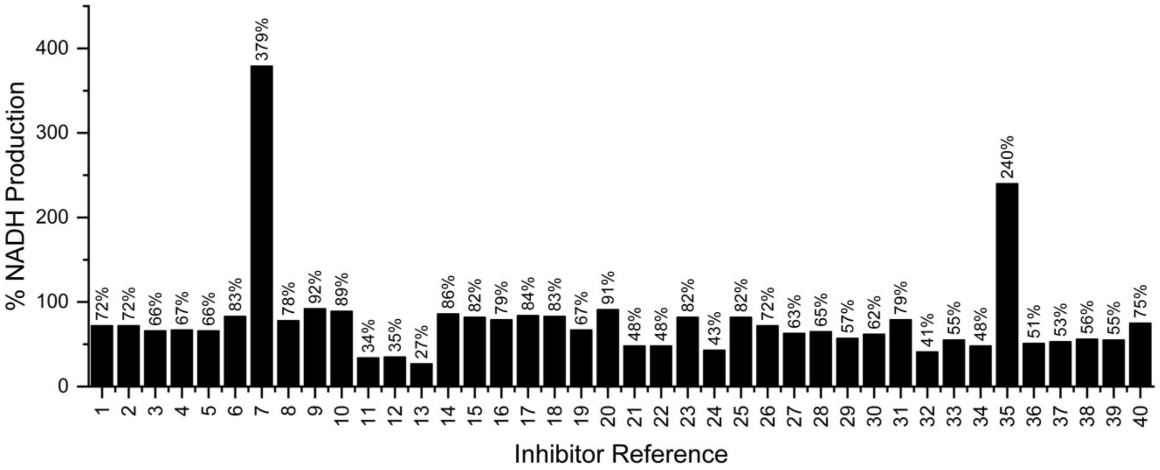

Figure S2. Bar chart comparing percentage NADH production in the presence of the 40 potential inhibitors hits. Percentage NADH production determined relative to the positive control containing no inhibitor. Inhibitors **7** & **35** demonstrate increased NADH production compared to the positive control.

3.2: Screening of 21 compounds following 1-hour preincubation with GMD

The assay was performed in 96-well flat bottomed, non-binding, polystyrene microtiter plates (Grenier 655906). NAD^+ (200 µM), inhibitor (50 µM) and GMD (50 µg/mL) were prepared in 50 mM sodium phosphate (pH 7.4) containing 0.5 mM $MgCl_2$ and 1 mM DTT. The samples were incubated at 4 °C for 1 hour before a solution of GDP-Man (final: 50 µM) was added to the plate and the fluorescence was measured at 25 °C for 60 minutes using a BMG labtech FLUOStar Omega microplate reader (excitation 355 nm; emission 460 nm). The limits of detection were analysed by control samples as followed: positive control contained no inhibitor; negative control contained no inhibitor or GDP-Man.
Data processed as previously described in section 3.1.

3.3: Inhibition Assay with Inhibitor 13

The assay was performed in 96-well flat bottomed, non-binding, polystyrene microtiter plates (Grenier 655906). Stock solution for each concentration of inhibitor **13** were prepared in DMSO and prepared following 2.5-fold serial dilution. Inhibitor (starting from final conc. 50 µM, 2.5-fold serial dilutions), NAD^+ (200 µM) and GMD (50 µg/mL) were prepared in 50 mM sodium phosphate (pH 7.4) containing 0.5 mM $MgCl_2$ and 1 mM DTT. The samples were incubated at 4 °C for 1 hour before a solution of GDP-Man (final: 50 µM) was added to the plate and the fluorescence was measured at 25 °C for 60 minutes using a BMG labtech FLUOStar Omega microplate reader (excitation 355 nm; emission 460 nm). The limits of detection were analysed by control samples as followed: positive control containing no inhibitor; negative control containing no inhibitor or GDP-Man.

Data Processing

All samples were analysed in triplicate. The error of each sample was calculated as standard error (equation S1) where n equals the samples size, x is the observed initial rate value for each sample and x̃ is the mean rate value for each sample.

$$standard\ error = \frac{\sqrt{\frac{\sum (x-\bar{x})^2}{(n-1)}}}{\sqrt{n}}$$

Equation S1: Standard error in initial rate obtained for each sample.

The initial rate of fluorescence increase was calculated over the first 20 minutes. The fluorescence was converted to % NADH production by comparison with the maximum and minimum values obtained from the positive and negative controls and errors were propagated accordingly. This data was then plotted against log (inhibitor concentration) for each sample in origin and the curve fitting was performed using the non-linear curve fit, using the Origin logistic function (equation S2), where A_1 is the curve's maximum, A_2 is the curve's minimum, x_0 is equal to the $IC_{50,}$ x is the log (inhibitor concentration), and p is the Hill slope parameter.

$$y = \frac{A_1 - A_2}{1 + (x/x_0)^p} + A_2$$

Equation S2: Equation for logistic curve fitting.

Section 4: Analysis of Inhibitor-GMD adducts by Mass Spectrometry

4.1: ESI-TOF Mass Spectrometry

GMD was buffer exchanged into 50 mM ammonium bicarbonate (pH 7.8) and concentrated to 0.3 mg/mL (6.3 µM) and incubated with inhibitor **13** (final 50 µM) at 4 °C overnight. An Agilent 1290 Infinity II series LC was used to inject 5 µl of sample into 5% MeCN (0.1% Formic Acid) at a flow rate of 0.6 mL/min and desalted inline using Agilent PLRP-S de-salt guard cartridges. This was eluted over 1 minute by 95% MeCN. The resulting mass/charge spectrum was analysed by an Agilent QTOF 6560, and deconvoluted using Agilent Masshunter BioConfirm Software between 5000-80,000 Da.

Table S6: Molecular weights for GDP-mannose dehydrogenase (protomer) and Inhibitor hit **13**.

	**Molecular Weight /Da**
**GDP-Mannose Dehydrogenase (GMD)**	47598.5
**Inhibitor 13**	446.41
**GMD + Inhibitor 13**	48044.9

GMD (No inhibitor)

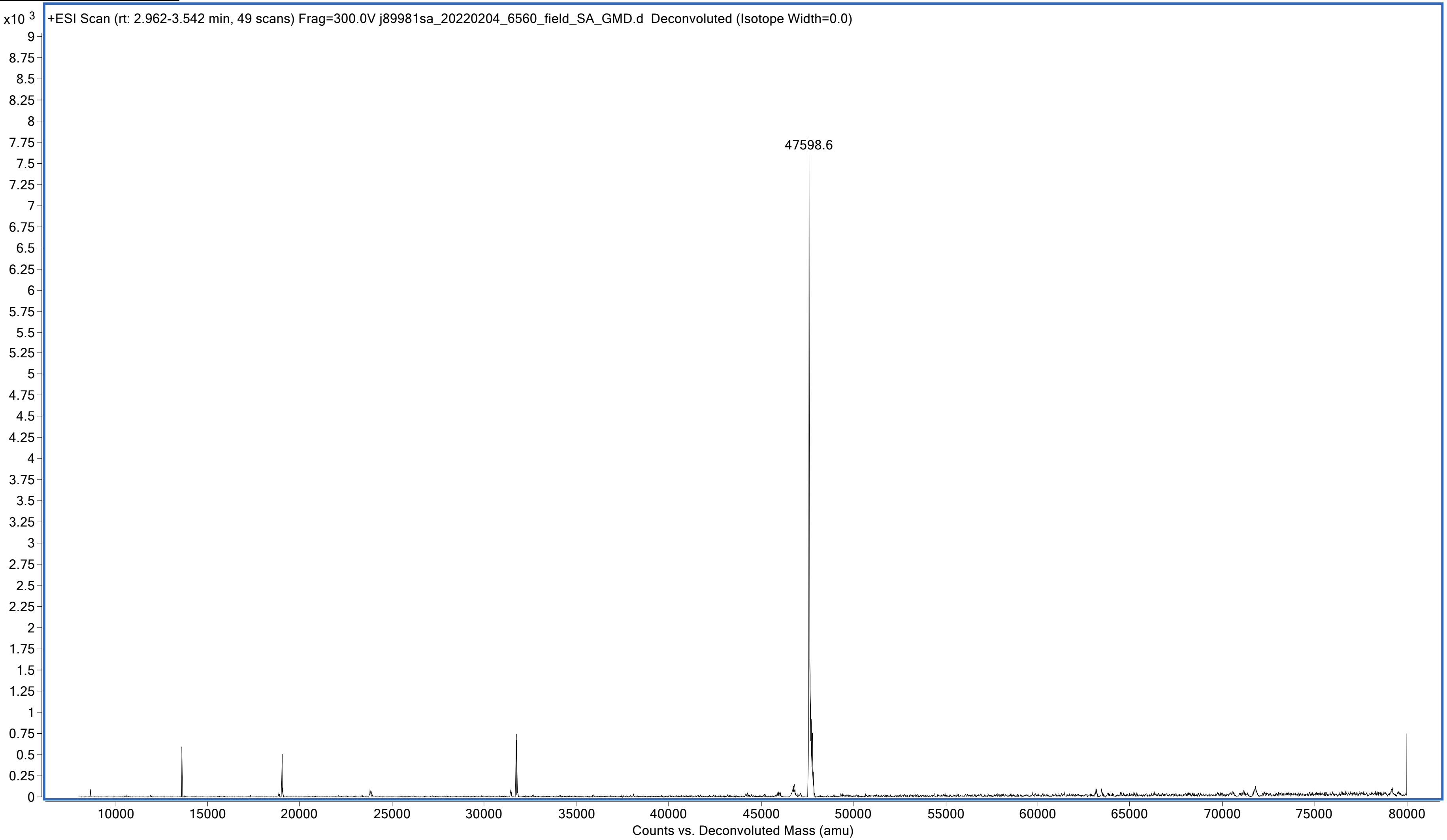

Figure S3: Deconvoluted protein-ESI-LCMS of GDP-mannose dehydrogenase (GMD)

GMD (No inhibitor, zoomed in)

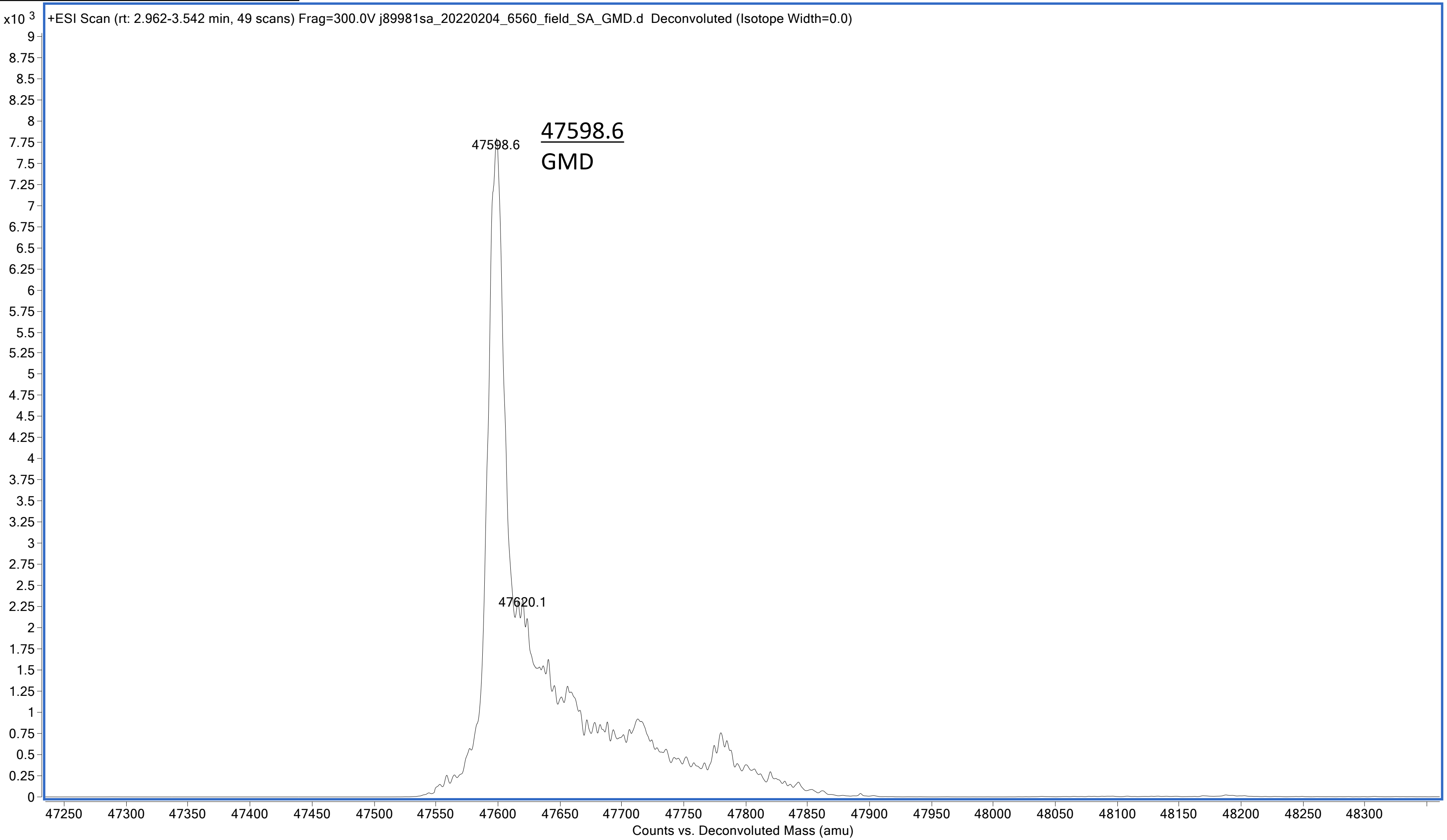

Figure S4: Deconvoluted protein-ESI-LCMS of GDP-mannose dehydrogenase (GMD) (zoomed in).

GMD + Inhibitor 13

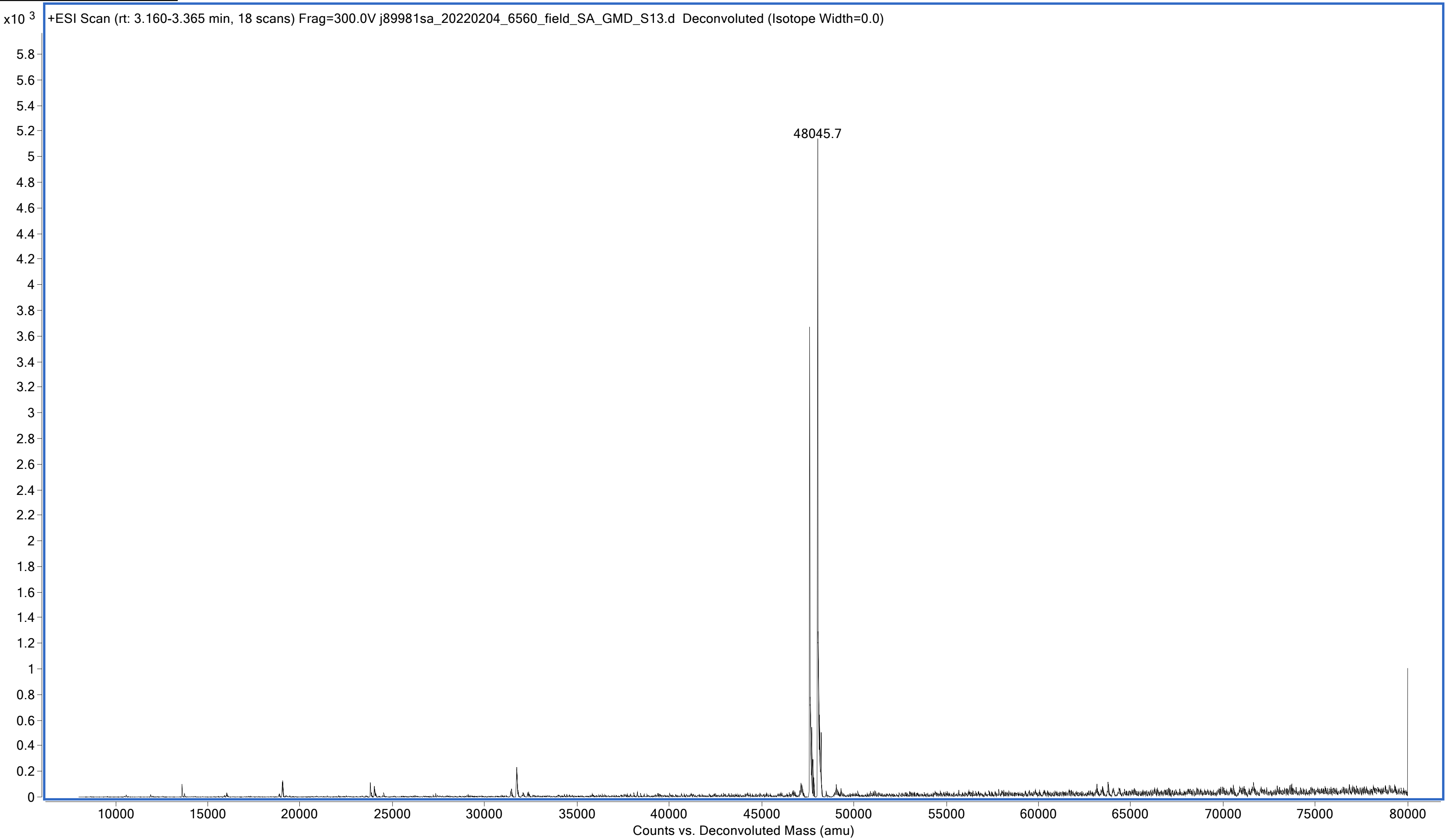

Figure S5: Deconvoluted protein-ESI-LCMS of GDP-mannose dehydrogenase (GMD) following overnight incubation with inhibitor **13**.

GMD + Inhibitor 13 (Zoom)

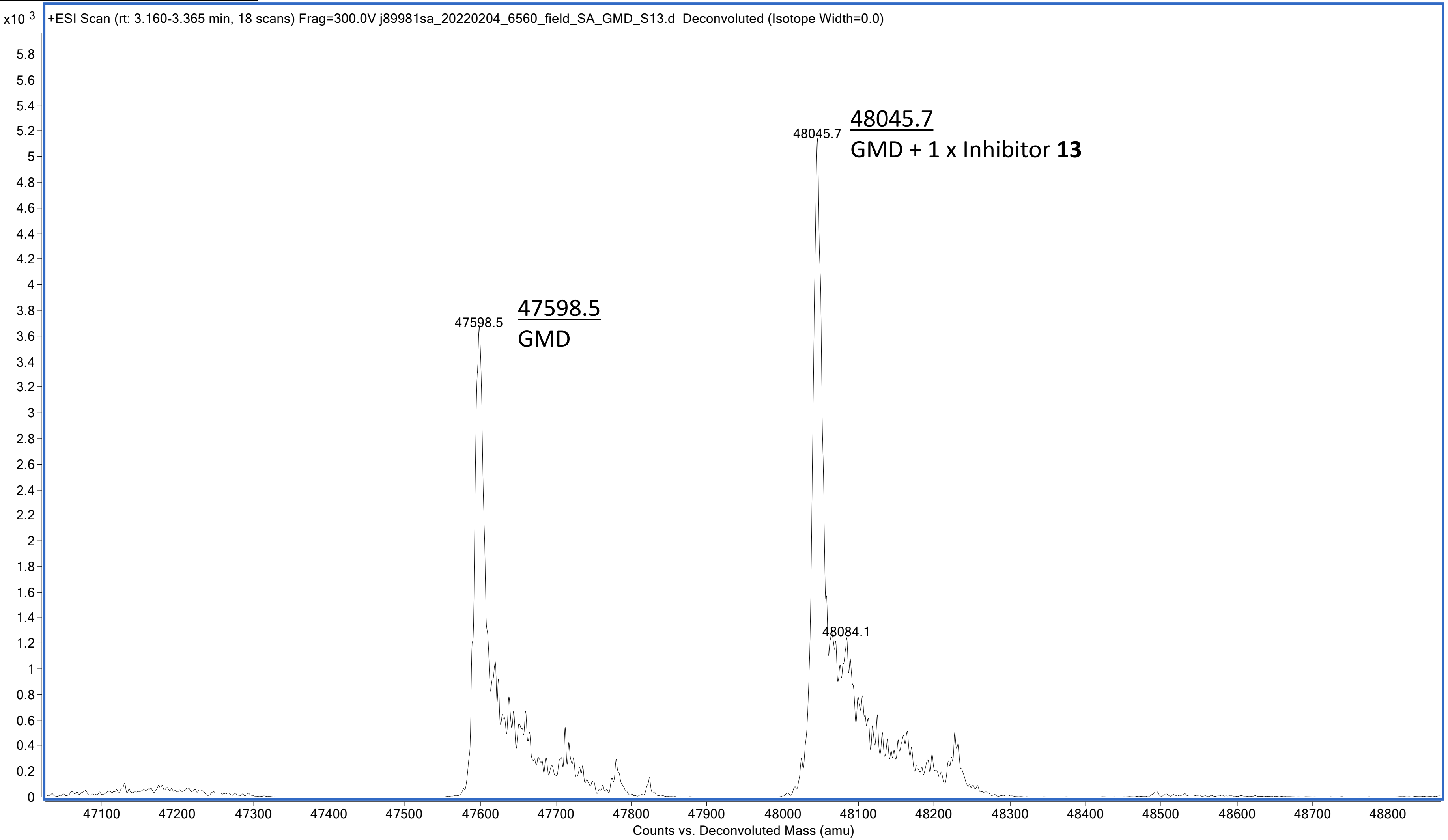

Figure S6: Deconvoluted protein-ESI-LCMS of GDP-mannose dehydrogenase (GMD) following overnight incubation with inhibitor **13** (zoomed in)

References

1 R. Bade, H.-F. Chan and J. Reynisson, *Eur J Med Chem*, 2010, **45**, 5646–5652.

2 R. A. Friesner, R. B. Murphy, M. P. Repasky, L. L. Frye, J. R. Greenwood, T. A. Halgren, P. C. Sanschagrin and D. T. Mainz, *J Med Chem*, 2006, **49**, 6177–6196.

3 M. McGann, *J Comput Aided Mol Des*, 2012, **26**, 897–906.

4 C. F. Snook, P. A. Tipton and L. J. Beamer, *Biochemistry*, 2003, **42**, 4658–4668.

5 H. M. Berman, J. Westbrook, Z. Feng, G. Gilliland, T. N. Bhat, H. Weissig, I. N. Shindyalov and P. E. Bourne, *Nucleic Acids Res*, 2000, **28**, 235–42.

6 G. M. Sastry, M. Adzhigirey, T. Day, R. Annabhimoju and W. Sherman, *J Comput Aided Mol Des*, 2013, **27**, 221–34.

7 E. Harder, W. Damm, J. Maple, C. Wu, M. Reboul, J. Y. Xiang, L. Wang, D. Lupyan, M. K. Dahlgren, J. L. Knight, J. W. Kaus, D. S. Cerutti, G. Krilov, W. L. Jorgensen, R. Abel and R. A. Friesner, *J Chem Theory Comput*, 2016, **12**, 281–296.

8 J. Cole, J. Willem M. Nissink and R. Taylor, 2005, pp. 379–415.

9 M. L. Verdonk, J. C. Cole, M. J. Hartshorn, C. W. Murray and R. D. Taylor, *Proteins: Structure, Function, and Bioinformatics*, 2003, **52**, 609–623.

10 G. Jones, P. Willett, R. C. Glen, A. R. Leach and R. Taylor, *J Mol Biol*, 1997, **267**, 727–748.

11 M. D. Eldridge, C. W. Murray, T. R. Auton, G. V. Paolini and R. P. Mee, *J Comput Aided Mol Des*, 1997, **11**, 425–445.

12 O. Korb, T. Stützle and T. E. Exner, *J Chem Inf Model*, 2009, **49**, 84–96.

13 W. T. M. Mooij and M. L. Verdonk, *Proteins*, 2005, **61**, 272–87.

14 K. Zhu, K. W. Borrelli, J. R. Greenwood, T. Day, R. Abel, R. S. Farid and E. Harder, *J Chem Inf Model*, 2014, **54**, 1932–1940.

15 O. V. Salomatina, N. S. Dyrkheeva, I. I. Popadyuk, A. L. Zakharenko, E. S. Ilina, N. I. Komarova, J. Reynisson, N. F. Salakhutdinov, O. I. Lavrik and K. P. Volcho, *Molecules*, 2021, **27**, 72.

16 A. A. Chepanova, N. S. Li-Zhulanov, A. S. Sukhikh, A. Zafar, J. Reynisson, A. L. Zakharenko, O. D. Zakharova, D. V. Korchagina, K. P. Volcho, N. F. Salakhutdinov and O. I. Lavrik, *Russ J Bioorg Chem*, 2019, **45**, 647–655.

17 T. Khomenko, A. Zakharenko, T. Odarchenko, H. J. Arabshahi, V. Sannikova, O. Zakharova, D. Korchagina, J. Reynisson, K. Volcho, N. Salakhutdinov and O. Lavrik, *Bioorg Med Chem*, 2016, **24**, 5573–5581.

18 Filimonov, Chepanova, Luzina, Zakharenko, Zakharova, Ilina, Dyrkheeva, Kuprushkin, Kolotaev, Khachatryan, Patel, Leung, Chand, Ayine-Tora, Reynisson, Volcho, Salakhutdinov and Lavrik, *Molecules*, 2019, **24**, 3711.

19 A. S. Filimonov, O. I. Yarovaya, A. V. Zaykovskaya, N. B. Rudometova, D. N. Shcherbakov, V. Yu. Chirkova, D. S. Baev, S. S. Borisevich, O. A. Luzina, O. V. Pyankov, R. A. Maksyutov and N. F. Salakhutdinov, *Viruses*, 2022, **14**, 2154.

20 O. Zakharova, O. Luzina, A. Zakharenko, D. Sokolov, A. Filimonov, N. Dyrkheeva, A. Chepanova, E. Ilina, A. Ilyina, K. Klabenkova, B. Chelobanov, D. Stetsenko, A. Zafar, C. Eurtivong, J. Reynisson, K. Volcho, N. Salakhutdinov and O. Lavrik, *Bioorg Med Chem*, 2018, **26**, 4470–4480.

21 L. E. Naught, S. Gilbert, R. Imhoff, C. Snook, L. Beamer and P. Tipton, *Biochemistry*, 2002, **41**, 9637–9645.
